# Quantitative Texture Analysis of Cervical Cytology Identifies Endometrial Lesions in Atypical Glandular Cells on Liquid-Based Cytology: A Pilot Study

**DOI:** 10.3390/diagnostics16040531

**Published:** 2026-02-10

**Authors:** Toshimichi Onuma, Akiko Shinagawa, Makoto Orisaka, Yoshio Yoshida

**Affiliations:** Department of Obstetrics and Gynecology, Faculty of Medical Sciences, University of Fukui, Fukui 910-1193, Japan; sngw@u-fukui.ac.jp (A.S.); orisaka@u-fukui.ac.jp (M.O.); yyoshida@u-fukui.ac.jp (Y.Y.)

**Keywords:** atypical glandular cells, computer-assisted, endometrial neoplasms, image processing, liquid-based cytology, Papanicolaou test, post-menopause

## Abstract

**Background/Objectives**: Within human papillomavirus (HPV)-based screening, cytology remains essential for cervical cancer detection while also potentially revealing endometrial pathology. This pilot study aimed to distinguish benign (normal) cases from atypical endometrial hyperplasia (AEH) and endometrial cancer (EC) within atypical glandular cell (AGC) cytology using quantitative analysis of liquid-based cervical cytology. **Methods**: SurePath and ThinPrep sets included 62 (37 normal, 25 AEH/EC) and 52 (24 normal, 28 AEH/EC) AGC cases, respectively. Semi-automatic QuPath analysis workflow detected cellular clusters; extracted texture, intensity, and geometric features; and produced case-level summaries. A random forest (RF) classifier was used to discriminate AEH/EC from normal cases. Feature subset selection was performed using a beam-search wrapper and joint hyperparameter tuning. Primary performance evaluation comprised stratified 5-fold cross-validation with metrics averaged across these folds. **Results**: Across both preparations, univariable analyses showed moderate discrimination overall which improved post-menopause. For SurePath and ThinPrep, the highest 10 areas under the curve (AUCs) were 0.701–0.773 (improving to 0.798–0.841 post-menopause) and 0.740–0.778 (improving to 0.832–0.884 post-menopause), respectively. Machine-learning RF models improved performance beyond univariable baselines. Cross-validated AUCs for SurePath and ThinPrep were 0.805 (95% confidence interval [CI], 0.683–0.927) and 0.887 (95% CI, 0.787–0.987), respectively. Features associated with higher AUCs differed between SurePath and ThinPrep, indicating platform-specific signals. **Conclusions**: Quantitative analysis of routine cervical cytology can augment expert reviews to help distinguish endometrial lesions among AGCs, particularly post-menopause. These software-based readouts can fit within existing workflows and may improve triage when morphology is subtle, including scenarios with HPV-negative screening results.

## 1. Introduction

Cervical cancer screening is increasingly shifting toward human papillomavirus (HPV) testing as the primary modality in line with contemporary guidelines and risk-based management frameworks [1,2]. Nevertheless, cytology retains the unique advantage of providing direct morphological assessment of epithelial lesions and continues to play a central role in clinical diagnosis using the Bethesda System [3]. Within this context, the identification of atypical glandular cells (AGCs) in cervical cytology is clinically significant as it necessitates prompt targeted follow-up and careful differential diagnosis [1,4]. AGCs represent a heterogeneous spectrum of findings, ranging from reactive changes to precancerous lesions and invasive malignancies across both squamous and glandular lineages, as described in contemporary reviews of glandular cytology and the Bethesda classification [5,6]. A previous study conducted in the United States has shown that approximately 5.2% of AGC cases have been associated with malignancy [4].

The incidence of endometrial cancer (EC) is rising worldwide, underscoring the need for improved detection strategies beyond traditional cervical pathways. Recent global estimates and trend analyses have shown substantial growth in EC burden since the 1990s, with 400,000 new cases reported worldwide annually in 2020–2022 [7,8,9]. Endometrial cell spillover into cervical cytology is clinically meaningful. In women aged ≥50 years, or after menopause, the presence of endometrial cells on Papanicolaou tests is associated with endometrial pathology, including carcinoma, and therefore warrants targeted evaluation [10,11]. Such cases are frequently categorized as AGCs, and EC represents 57.6% of AGC-associated malignancies [4]. These observations suggest the need for diagnostic approaches that carefully consider both endometrial and cervical sources of disease. However, consistent visual appraisal is difficult [12,13,14]. Moreover, reliable distinction among these diverse etiologies remains challenging, as conventional cytomorphologic interpretation is subject to observer variability [15,16]. Therefore, advanced imaging analytics may detect endometrial cell leakage and contribute to the diagnosis of EC.

Recent advances in digital pathology and image analysis enable reproducible extraction and quantification of diverse features, such as shape, texture, and spatial arrangement, through generating standardized measurements from whole-slide and cytology images [17,18,19]. These computational methods can complement traditional experience-dependent assessments through revealing subtle differences not readily appreciable to an unaided microscopy [12,20]. Prior studies and systematic reviews of automated cervical screening tools have reported improvements in both accuracy and efficiency when using artificial intelligence (AI)-assisted digital cytology [21,22].

Within this background, this pilot study aims to distinguish benign (normal) cases from atypical endometrial hyperplasia (AEH) and EC within AGC cytology using quantitative image analysis of liquid-based cervical cytology.

## 2. Materials and Methods

### 2.1. Case Selection

Between 2014 and 2024, we identified 161 cases of AGCs at the University of Fukui Hospital in which liquid-based cervical cytology (LBC) had been used (SurePath, Becton, Dickinson and Company, Franklin Lakes, NJ, USA; or ThinPrep, Hologic, Inc., Marlborough, MA, USA). The AGC subcategories were not routinely assigned [23]. The platform varied according to period—SurePath (2014–2017), ThinPrep (2018–2023), and SurePath again in 2024—reflecting transitions prompted by institutional equipment upgrades. Reference diagnoses were determined from histologic assessment or follow-up cytology. Histological diagnosis was derived from biopsy or surgical specimens. The benign (normal) category included the following: (i) cases with benign histologic findings and no evidence of atypical endometrial hyperplasia or endometrial cancer (AEH/EC) and/or (ii) cases in which repeat cervical cytology during follow-up was negative for intraepithelial lesion or malignancy without a subsequent clinical diagnosis of endometrial neoplasia. Trained pathology specialists performed all the histopathologic and cytologic assessments. In this study, AEH/EC comprised atypical endometrial hyperplasia and endometrial cancer, including endometrioid carcinoma and non-endometrioid histologies such as serous and clear cell carcinoma.

This retrospective study was approved by the Research Ethics Committee of the University of Fukui (Approval No. 20250063). Written consent was waived and opt-out consent was implemented via public notice on the following institutional website: https://research.hosp.u-fukui.ac.jp/rinsho/ethicscommittee/koukai_list/#sankafujinka (accessed on 1 December 2025).

**Figure 1:**
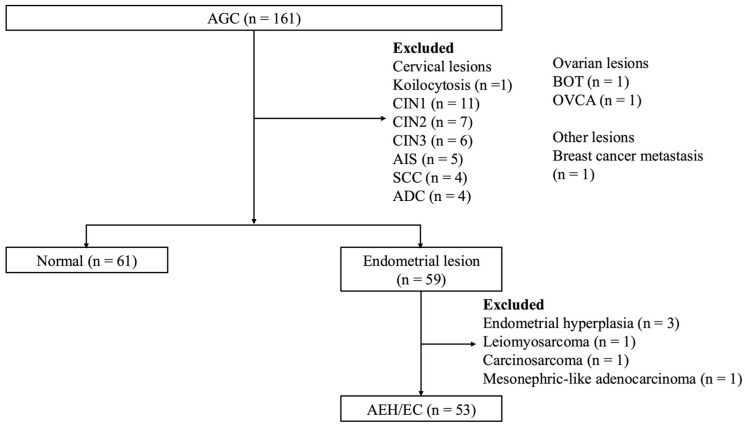
Pathological outcomes of cases with AGC on cervical cytology. Final diagnoses were categorized as normal (*n* = 61) or AEH/EC (*n* = 53). ADC, adenocarcinoma; AEH/EC, atypical endometrial hyperplasia or endometrial cancer; AGC, atypical glandular cells; AIS, adenocarcinoma in situ; BOT, borderline ovarian tumor; CIN1, cervical intraepithelial neoplasia 1; CIN2, cervical intraepithelial neoplasia 2; CIN3, cervical intraepithelial neoplasia 3; OVCA, ovarian cancer; SCC, squamous cell carcinoma.

Figure 1 shows the pathological results of the AGC cases, of which 36.6% (59/161) were lesions of endometrial origin and 37.9% (61/161) were classified as normal. On the SurePath slides, 37 cases were classified as normal (median age, 48.3 [range, 31.6–74.0] years) and 25 were classified as AEH/EC (median age, 58.6 [range, 41.1–81.3] years). Within the AEH/EC group, there were two cases of AEH, while ECs comprised endometrioid grade 1 (*n* = 12), grade 2 (*n* = 7), and grade 3 (*n* = 2); serous carcinoma (*n* = 1); and clear cell carcinoma (*n* = 1). Among the post-menopausal subset, 13 cases were normal and 17 cases were AEH/ECs, comprising endometrioid grade 1 (*n* = 8), grade 2 (*n* = 6), and grade 3 (*n* = 1); serous carcinoma (*n* = 1); and clear cell carcinoma (*n* = 1).

On the ThinPrep slides, 24 cases were classified as normal (median age, 41.5 [range, 21.2–80.5] years) and 28 were classified as AEH/EC (median age, 59.5 [range, 35.2–79.8] years). Within the AEH/EC group, there was one case of AEH, and the remaining carcinomas included endometrioid grade 1 (*n* = 13), grade 2 (*n* = 6), and grade 3 (*n* = 4); serous carcinoma (*n* = 2); and clear cell carcinoma (*n* = 2). Among post-menopausal patients, there were 5 normal cases and 19 AEH/EC cases, comprising endometrioid grade 1 (*n* = 8), grade 2 (*n* = 5), and grade 3 (*n* = 3); serous carcinoma (*n* = 2); and clear cell carcinoma (*n* = 1).

### 2.2. Image Acquisition and Preprocessing

**Figure 2:**
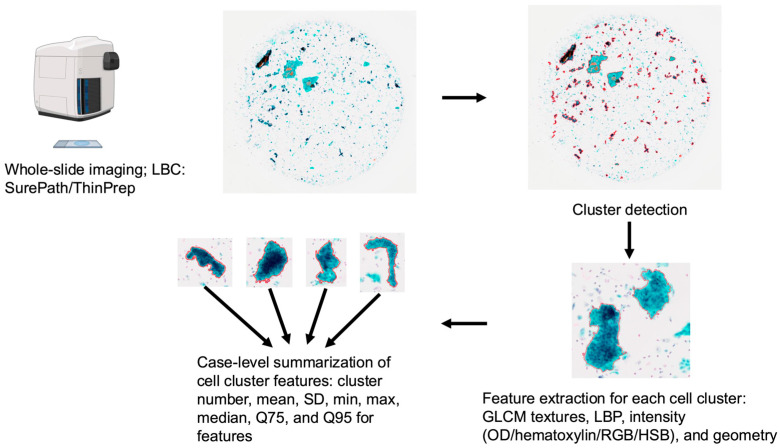
Workflow for quantitative analysis of AGC-classified liquid-based cervical cytology. Whole-slide images from LBC (SurePath or ThinPrep) were digitized and preprocessed. Cell clusters were automatically detected on downsampled images and mapped back to full-resolution coordinates. For each detected cluster, quantitative readouts were extracted from regions of interest (texture, intensity, and geometric descriptors). Case-level data were then generated through summarizing cluster-wise measurements (cluster count and distributional statistics: mean, SD, minimum, maximum, median, Q75, and Q95). These aggregated outputs formed the inputs for subsequent diagnostic analyses. GLCM, gray-level co-occurrence matrix; HSB, hue, saturation, brightness; LBC, liquid-based cytology; LBP, local binary pattern; max, maximum; min, minimum; OD, optical density; Q75, 75th percentile; Q95, 95th percentile; RGB, red, green, blue; SD, standard deviation.

The analytical workflow is illustrated in Figure 2. Whole-slide images, which were acquired using a SLIDEVIEW VS200 research slide scanner (Evident, Tokyo, Japan), were then processed semi-automatically using QuPath and OpenCV to detect and record cell clusters [24]. The slides were initially scanned at one-third resolution, after which coordinates were adjusted to full scale. Red, green, and blue (RGB) images were converted to grayscale, and global thresholding (fixed threshold = 210) was applied to binarize the images. Contours of connected foreground regions were then extracted and their areas were calculated. To reduce false detections, only regions with areas between 10,000 and 100,000,000 pixels in the downsampled images were retained as cell clusters. These regions were polygonized, rescaled to original slide coordinates, and saved as annotations in QuPath. Any enclosed areas that did not represent cell clusters were manually excluded (Figure 2).

### 2.3. Feature Extraction

**Figure 3:**
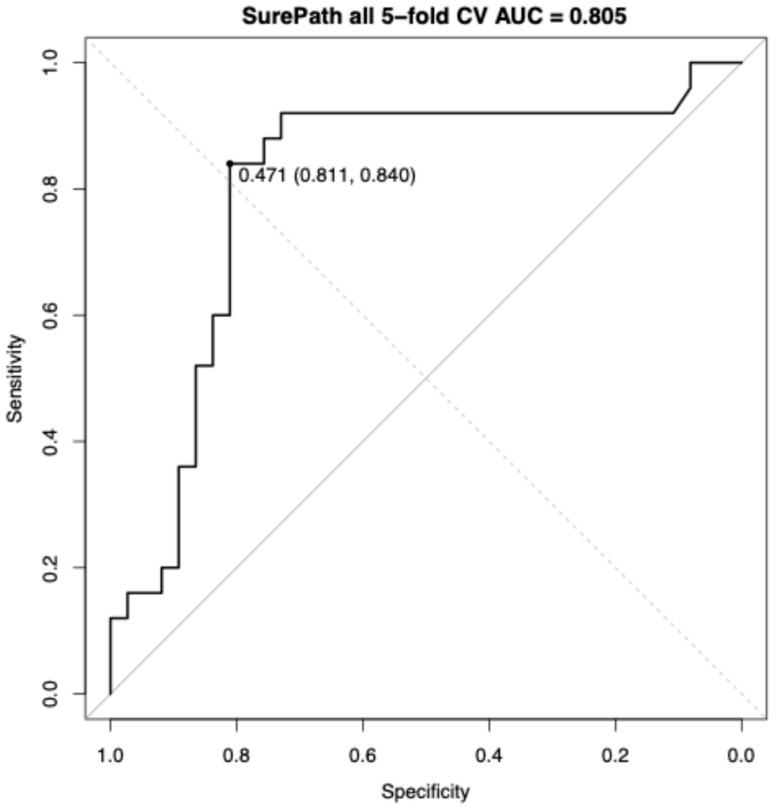
ROC curve for AGC cases on SurePath with 5-fold cross-validation. ROC curve analysis for distinguishing AEH/EC from normal within AGC cases on SurePath cytology. Performance was estimated using stratified 5-fold cross-validation with out-of-fold predictions. The CV AUC was 0.805. AEH/EC, atypical endometrial hyperplasia or endometrial cancer; AGC, atypical glandular cells; CV AUC, cross-validated area under the curve; ROC, receiver operating characteristic.

The following features were measured: (i) gray-level co-occurrence matrix (GLCM)-based texture features, including correlation, contrast, homogeneity, energy, entropy, difference entropy/variance, sum average, sum variance, and sum of squares variance, along with their normalized counterparts; (ii) local binary pattern (LBP) features, including mean, standard deviation (SD), and entropy [25,26]; and (iii) intensity statistics within regions of interest sampled at 2.00 µm per pixel, including mean, SD, minimum, maximum, median values for optical density (OD) sum, hematoxylin, residual, RGB channels, hue, saturation, and brightness. Geometric descriptors (area, µm^2^; length, µm) were also recorded (Figure 3). For each case, the mean, SD, maximum, minimum, median, and 75th (Q75) and 95th (Q95) percentiles of these measurements were calculated (Figure 3). One case containing only a single cluster in SurePath was excluded because the SD could not be defined. Summaries of the extracted data are presented in Supplementary Tables S1 and S2.

### 2.4. Statistical Analyses

All statistical computations were performed using EZR version 1.42 software [27]. Receiver operating characteristic (ROC) curves were constructed for each feature and the area under the curve (AUC) was used as the primary univariable metric. The Youden index was applied to determine the optimal threshold and the diagnostic performance for AEH/EC was calculated accordingly. Multivariable discrimination was assessed using a random forest (RF) classifier [28,29]. Feature subset selection was performed using a beam-search wrapper that increased subset size stepwise to identify compact feature panels [30,31,32]. For each candidate subset, an RF model was trained with joint tuning of the number of trees, mtry, minimal node size, and split rule. Class weights were set inverse to class frequencies. The final feature subset and hyperparameters were selected by maximizing performance under stratified 5-fold cross-validation (CV) [33]. Beam search and simultaneous RF tuning was conducted within the training folds to prevent information leakage [34]. Model performance was estimated exclusively from out-of-fold (OOF) predictions aggregated across these 5 folds [33]. From these OOF scores, the ROC curves, the cross-validated AUC (CV AUC) with DeLong 95% confidence intervals (CIs), and operating characteristics at the Youden point were derived.

## 3. Results

### 3.1. ROC Curve Analysis Between Normal and Endometrial Lesion Groups Using SurePath

Table 1 presents the ROC curve analysis for diagnosing AEH/EC using SurePath. In AEH/EC cases, the median cell cluster count was 52 (range, 1–1797), whereas in normal cases, the median cell cluster count was 58 (range, 1–1743). In univariate ROC curve analyses, dispersion-type metrics provided the strongest discrimination. Hematoxylin variability performed the best (AUC, 0.773), followed by residual variability and summed OD. Intensity features showed moderate performance, including minimum of blue, maximum; minimum of residual, maximum; and hue, mean. Red-channel dispersion occurred between these groups. Across all markers, the AUCs ranged from 0.701 to 0.773.

Performance improved in the post-menopausal subgroup where dispersion-type features again predominated. Red-channel variability exhibited the highest accuracy (SD of red, median; AUC, 0.841). Hematoxylin intensities were comparably strong (minimum of hematoxylin, median; AUC, 0.833; minimum of hematoxylin, mean; AUC, 0.819). A GLCM contrast measure captured additional heterogeneity (maximum of normalized difference entropy; AUC, 0.814). Sensitivity learning behavior was evident for blue intensity (minimum of blue, maximum; sensitivity, 0.941). Overall, the AUCs ranged from 0.798 to 0.841, indicating uniformly stronger separation post-menopause.

### 3.2. RF-Driven Feature Subsets and ROC Performance in SurePath

Using the locked three-feature subset (minimum of hematoxylin, SD; SD of hematoxylin, SD; and minimum of red, SD), and tuned RF hyperparameters, a stratified 5-fold cross-validation yielded a CV AUC of 0.805 (95% CI, 0.683–0.927). At the Youden threshold (0.471), sensitivity was 0.840 and specificity was 0.811 (Figure 3).

### 3.3. ROC Curve Analysis Between Normal and Endometrial Lesion Groups Using ThinPrep

Table 2 presents the ROC curve performance of ThinPrep in diagnosing AEH/EC. In AEH/EC cases, the median cell cluster count was 347 (range, 6–1351), whereas in normal cases, the median cell cluster count was 267 (range, 26–2007). In ThinPrep-based univariate ROC curve analyses, the overall discrimination was moderate with AUCs ranging from 0.728 to 0.778. Among all features, GLCM variance-type metrics performed the best, with the minimum sum of squares variance and minimum sum variance each achieving an AUC of 0.778 with identical operating points. Intensity markers followed closely. The minimum of OD sum: median reached an AUC of 0.765 while the minimum of hematoxylin: median achieved an AUC of 0.764, favoring specificity (specificity, 0.958). Dispersion-oriented measures clustered just below these highest performers, including the SD of brightness: median; the SD of brightness: mean; the SD of residual: minimum; minimum of residual: maximum; the SD of red: minimum; and the SD of residual: maximum.

Performance improved markedly in the post-menopausal subgroup with AUCs concentrated in a higher band (range, 0.832–0.884). The SD of hematoxylin: mean yielded the highest accuracy (AUC, 0.884; sensitivity, 0.789; specificity, 1.000). Several features demonstrated uniformly high sensitivity and specificity at Youden optimal thresholds, including the minimum of sum of squares variance, minimum of sum variance (each AUC, 0.842; sensitivity, 1.000; specificity, 0.800), and minimum of homogeneity (AUC, 0.842; sensitivity, 0.737; specificity, 1.000). Consistent with this finding, slightly lower AUCs with perfect specificity were observed for the SD of OD sum: median, as well as for Q95 of hematoxylin: SD, indicating uniformly stronger separation post-menopause.

### 3.4. RF-Driven Feature Subsets and ROC Performance in ThinPrep

**Figure 4:**
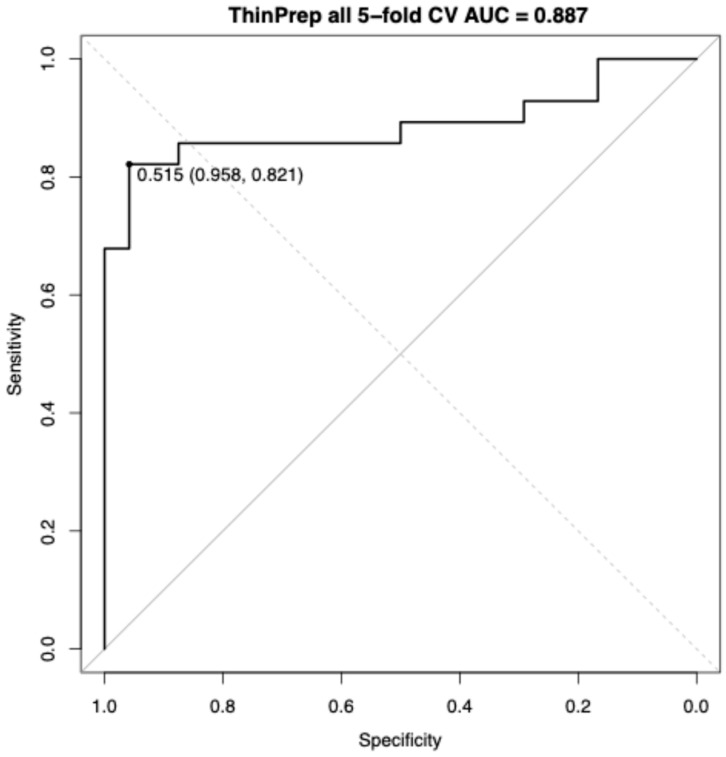
ROC curve for AGC cases on ThinPrep with 5-fold cross-validation. ROC curve analysis for distinguishing AEH/EC from normal within AGC cases on ThinPrep cytology. Performance was estimated using stratified 5-fold cross-validation with out-of-fold predictions. The CV AUC was 0.887. AEH/EC, atypical endometrial hyperplasia or endometrial cancer; AGC, atypical glandular cells; CV AUC, cross-validated area under the curve; ROC, receiver operating characteristic.

Using the locked five-feature subset (SD of brightness, median; minimum of green, minimum; minimum of hematoxylin, median; mean of length; and minimum of sum of squares variances) and tuned RF hyperparameters, a stratified 5-fold cross-validation yielded a CV AUC of 0.887 (95% CI, 0.787–0.987). At the Youden threshold (0.515), sensitivity was 0.821 and specificity was 0.958 (Figure 4).

## 4. Discussion

Across SurePath and ThinPrep liquid-based preparations, univariate ROC analyses showed only moderate discrimination between normal cases and AEH/EC cases overall, but consistently stronger separation post-menopause. In SurePath, single-marker AUCs ranged from 0.701 to 0.773 and shifted upward to 0.798–0.841 post-menopause. A compact RF model trained on the overall SurePath cohort achieved a stratified 5-fold CV AUC of 0.805 (95% CI, 0.683–0.927). In ThinPrep, overall univariate AUCs were 0.728–0.778, concentrating at 0.832–0.884 post-menopause with the highest marker at 0.884. The overall ThinPrep RF yielded a stratified 5-fold CV AUC of 0.887 (95% CI, 0.787–0.987). In the univariable analyses, the highest AUC features differed between SurePath and ThinPrep. Similarly, in RF models, the feature sets selected also differed across the two preparations.

Across both preparations, AEH/EC could be differentiated from normal cases. Univariate ROC analyses indicated moderate separability overall with stronger separation post-menopause, and compact RF models further improved performance. This was consistent with the prior clinical literature indicating that a subset of AGC has shown cytologic features suggestive of AEH/EC and has been associated with a clinically meaningful risk of underlying neoplasia [4,5,6]. The stronger separation observed after menopause was clinically plausible, as reports have shown that endometrial cell findings on cervical cytology in older or post-menopausal women were associated with EC [10,11]. These findings suggested that quantitative image features can assist where human visual assessment can be challenging and offer operational leverage, particularly given the interobserver variability in AGC interpretation [15,16]. RGB-derived values should not be equated with pure hematoxylin/eosin concentrations without stain unmixing or normalization [35]. Preparation- and scanner-related variability also indicated the need for harmonization in future external validation [36]. If these limitations can be overcome, image analysis may extend diagnostic capability in selected contexts.

ThinPrep and SurePath shared high-ranking features, yet the specific highest markers differed, indicating a preparation-dependent appearance that must be considered in an image analysis workflow. Cytomorphologic comparisons indicated systematic platform-dependent differences in cellularity, cluster architecture, and background characteristics [37,38,39]. ThinPrep tended to yield flatter and more fragmented cell aggregates, whereas SurePath more often produced larger and more three-dimensional aggregates [37,38,39]. These preparation-related differences likely influenced which features were most informative for classification on each platform. Furthermore, one comparative screening study reported unequal unsatisfactory rates and operational behaviors between the two systems [38]. Taken together, these data could suggest the need for protocol-specific preprocessing/feature selection, stain normalization, and cross-preparation validation to ensure transferability in line with evidence that stain/scanner variability can degrade model performance and should be mitigated during external validation [36,40].

Image analysis-based adjuncts were technically feasible and increasingly validated. AI-assisted or computer-aided cytology can triage slides at scale, improving sensitivity while maintaining clinically acceptable specificity in both population screening and prospective evaluations [41,42,43,44]. These software-based methods can analyze quantitative morphology, intensity, and texture features from standard liquid-based preparations. These image-based measures can offer objective and reproducible aids during routine review. Because primary HPV screening is designed to detect cervical diseases, endometrial lesions such as AEH/EC can fall outside the intended detection scope of HPV testing and may therefore be overlooked, even though cervicovaginal cytology has historically identified a substantial proportion of AEH/EC, albeit incidentally [2,45]. In this context, our results can extend to prior AI-cytology work that has largely focused on cervical lesion detection and grading [22,41,42,43,44] by evaluating AEH/EC discrimination within AGC, a clinically difficult diagnostic setting.

This approach may be most useful as a decision support adjunct in AGC cases with subtle or equivocal morphology, and it may help identify AEH/EC that could otherwise be missed in HPV-negative cases under HPV primary screening.

This study has some limitations. It was a single-center retrospective study without external validation and the reported performance reflects internal cross-validation only. Therefore, external multi-site validation is required. However, the cohort reflected a real-world clinical workflow in which liquid-based cervical cytology cases classified as AGC by trained specialists were managed in routine practice, supporting the practical relevance of the findings. Owing to the limited number of cases, RF-based analyses were not performed within the post-menopausal subgroup. Nevertheless, the consistently stronger discrimination observed after menopause could suggest a clinically relevant subgroup and provide a clear rationale for targeted validation. Textural features may be influenced by pre-analytical and analytical factors, including staining procedures, scanner calibration, and platform architecture. Because SurePath and ThinPrep were used in temporally separated periods, cross-platform differences may be confounded by changes in case mix. Therefore, our analyses were intended primarily as within-platform evaluations, and any cross-platform comparisons should be interpreted cautiously. Importantly, the inclusion of both SurePath and ThinPrep may help to clarify preparation-dependent behavior and may indicate the need for standardization. Thus, prospective multicenter validation with standardized workflows will be necessary.

## 5. Conclusions

Quantitative analysis of liquid-based cervical cytology distinguishing endometrial lesions across both SurePath and ThinPrep with compact machine-learning models provided clear gains over univariate baselines. These model-based readouts can complement expert reviews and, in some cases where morphology can be subtle, may extend diagnostic capabilities beyond the limits of unaided visual inspections. Taken together, the findings can support a practical, prespecified RF panel as a software adjunct to HPV primary screening, including the potential to help identify ECs in HPV-negative cases.

## Figures and Tables

**Table 1:** Highest 10 ROC curve analysis results between the normal and AEH/EC lesion groups using SurePath.

Variable	AUC	Sensitivity	Specificity	Cutoff	AEH/EC
SD of hematoxylin: SD	0.773	0.500	0.944	0.158	LOW
SD of residual: SD	0.762	0.500	0.917	0.077	LOW
SD of OD sum: SD	0.757	0.875	0.556	0.227	LOW
Min. of blue: max.	0.726	0.720	0.730	216.500	HIGH
SD of red: median	0.721	0.625	0.750	35.262	LOW
SD of red: mean *	0.706	0.625	0.694	29.240	LOW
SD of red: mean *	0.706	0.542	0.778	27.140	LOW
Min. of residual: max.	0.705	0.480	0.865	0.446	HIGH
Max. of red: min.	0.705	0.800	0.622	126.500	LOW
Min. of hue: mean	0.701	0.800	0.676	0.005	HIGH
SurePath menopause cases					
SD of red: median	0.841	0.813	0.846	35.262	LOW
Min. of hematoxylin: median	0.833	0.824	0.769	0.122	HIGH
Min. of Hematoxylin: Mean	0.819	0.765	0.769	0.186	HIGH
SD of residual: max.	0.817	0.750	0.846	0.224	LOW
Max. of normalized difference entropy	0.814	0.706	0.923	0.997	LOW
Min. of blue: max.	0.810	0.941	0.615	212.000	HIGH
Max. of red: mean	0.805	0.706	0.846	158.892	LOW
Max. of red: median	0.801	0.765	0.846	172.000	LOW
SD of hematoxylin: max.	0.798	0.750	0.846	0.431	LOW
SD of red: mean	0.798	0.750	0.769	27.439	LOW

AEH/EC, atypical endometrial hyperplasia or endometrial cancer; AUC, area under the curve; OD, optical density; max., maximum; min., minimum; ROC, receiver operating characteristic; SD, standard deviation. * In some cases, multiple thresholds yielded identical maximum Youden index values.

**Table 2:** Highest 10 results of ROC curve analysis between the normal and AEH/EC lesion groups using ThinPrep.

ThinPrep, All Cases	AUC	Sensitivity	Specificity	Cutoff	AEH/EC
Min. of sum of squares variance	0.778	0.893	0.667	0.487	HIGH
Min. of sum variance	0.778	0.893	0.667	0.487	HIGH
Min. of OD sum: median	0.765	0.786	0.667	0.294	HIGH
Min. of hematoxylin: median	0.764	0.607	0.958	0.109	HIGH
SD of brightness: median	0.757	0.714	0.708	1184.232	HIGH
SD of brightness: mean	0.756	0.821	0.708	0.090	LOW
SD of residual: min.	0.751	0.786	0.708	0.079	LOW
Min. of residual: max. *	0.750	0.821	0.708	0.079	LOW
SD of red: min.	0.750	0.679	0.792	0.131	HIGH
Min. of residual: max. *	0.740	0.857	0.583	17.848	LOW
ThinPrep, menopause cases					
SD of hematoxylin: mean	0.884	0.789	1.000	0.366	LOW
Min. of sum of squares variance	0.842	1.000	0.800	0.486	HIGH
Min. of sum variance	0.842	1.000	0.800	0.486	HIGH
Min. of homogeneity	0.842	0.737	1.000	0.109	HIGH
Q75 of OD sum: SD	0.842	0.737	1.000	0.410	HIGH
Min. of hematoxylin: median	0.842	0.737	1.000	0.899	LOW
SD of OD sum: mean	0.832	0.842	0.800	0.537	LOW
SD of OD sum: median	0.832	0.737	1.000	0.409	LOW
Q95 of hematoxylin: SD	0.832	0.737	1.000	0.511	LOW
SD of hematoxylin: median	0.832	0.684	1.000	0.960	LOW

AEH/EC, atypical endometrial hyperplasia or endometrial cancer; max., maximum; min., minimum; OD, optical density; Q75, 75th percentile; Q95, 95th percentile; SD, standard deviation. * In some cases, multiple thresholds yielded identical maximum Youden index values.

## Data Availability

The data are available from the corresponding author upon reasonable request and with approval from the Research Ethics Committee of University of Fukui.

## Supplementary Materials

The following supporting information can be downloaded at: https://www.mdpi.com/article/10.3390/diagnostics16040531/s1, Table S1: Summaries of the extracted data in SurePath; Table S2: Summaries of the extracted data in ThinPrep.

## Abbreviations

The following abbreviations are used in this manuscript:

ADC	Adenocarcinoma
AEH	Atypical endometrial hyperplasia
AEH/EC	Atypical endometrial hyperplasia/endometrial cancer
AGCs	Atypical glandular cells
AIS	Adenocarcinoma in situ
BOT	Borderline ovarian tumor
CI	Confidence interval
CIN	Cervical intraepithelial neoplasia
CV	Cross-validation
CV AUC	Cross-validated area under the curve
EC	Endometrial cancer
GLCM	Gray-level co-occurrence matrix
HPV	Human papillomavirus
HSB	Hue, saturation, brightness
LBC	Liquid-based cytology
Max.	Maximum
Min.	Minimum
OD	Optical density
OOF	Out-of-fold
OVCA	Ovarian cancer
Q75	75th percentile
Q95	95th percentile
RF	Random forest
RGB	Red, green, blue
ROC	Receiver operating characteristic
SCC	Squamous cell carcinoma
SD	Standard deviation
